# Improved Algal Toxicity Test System for Robust *Omics*-Driven Mode-of-Action Discovery in *Chlamydomonas reinhardtii*

**DOI:** 10.3390/metabo9050094

**Published:** 2019-05-10

**Authors:** Stefan Schade, Emma Butler, Steve Gutsell, Geoff Hodges, John K. Colbourne, Mark R. Viant

**Affiliations:** 1 School of Biosciences, University of Birmingham, Edgbaston, Birmingham B15 2TT, UK; sxs1318@bham.ac.uk (S.S.); J.K.Colbourne@bham.ac.uk (J.K.C.); 2 Safety and Environmental Assurance Centre, Unilever, Colworth Science Park, Sharnbrook MK44 1LQ, UK; Emma.Butler@unilever.com (E.B.); Steve.Gutsell@unilever.com (S.G.); Geoff.Hodges@unilever.com (G.H.)

**Keywords:** synchronisation, algae, bioassay, biomarker, key event, adverse outcome pathway

## Abstract

Algae are key components of aquatic food chains. Consequently, they are internationally recognised test species for the environmental safety assessment of chemicals. However, existing algal toxicity test guidelines are not yet optimized to discover molecular modes of action, which require highly-replicated and carefully controlled experiments. Here, we set out to develop a robust, miniaturised and scalable *Chlamydomonas reinhardtii* toxicity testing approach tailored to meet these demands. We primarily investigated the benefits of synchronised cultures for molecular studies, and of exposure designs that restrict chemical volatilisation yet yield sufficient algal biomass for omics analyses. Flow cytometry and direct-infusion mass spectrometry metabolomics revealed significant and time-resolved changes in sample composition of synchronised cultures. Synchronised cultures in sealed glass vials achieved adequate growth rates at previously unachievably-high inoculation cell densities, with minimal pH drift and negligible chemical loss over 24-h exposures. Algal exposures to a volatile test compound (chlorobenzene) yielded relatively high reproducibility of metabolic phenotypes over experimental repeats. This experimental test system extends existing toxicity testing formats to allow highly-replicated, *omics*-driven, mode-of-action discovery.

## 1. Introduction

Algae are internationally established test organisms in chemical risk assessment [1,2,3]. Toxicity test guidelines incorporating algae are largely focused on traditional apical endpoints such as growth rates following a 72-h chemical exposure. By consequence, no knowledge is obtained on the causes of chemical toxicity, nor on the generality of the chemical effects on other species. Increasingly, chemical risk assessment includes New Approach Methodologies (NAMs) to improve precision in the prioritisation and chemical categorisation of chemicals [4]. One application of NAMs is to increase confidence in cause–effect analyses by providing mechanistic evidence on the harmful effects of chemicals, e.g., in the form of rapid and large-scale discovery of molecular key events (mKE) in an adverse outcome pathway (AOP) framework [5]. This transition, from traditional whole organism endpoint-based to molecular pathway-based (eco)toxicology, is motivated by the application of high-content technologies such as transcriptomics and metabolomics, which have demonstrated a capability to discover biological processes [6]. A robust, scalable test guideline for *omics*-driven discovery of toxicological key events in algae first requires a rigorously characterised algae culturing and exposure protocol with biological processes synchronised across all cells within a test population, so to minimise molecular variation for the pathway-based cause-effect assessments. Ideally, such an exposure system features minimal test duration to facilitate rapid measurements, and is matched to an algal lifecycle to allow accurate linking of the molecular perturbations to growth inhibition to address regulatory requirements of phenotypic anchoring [6,7,8,9,10]. Additionally, the exposure system should be capable of testing both non-volatile and volatile toxicants, and providing highly-replicated generation of sufficient algal biomass for multi-*omics* measurements. Design of such a robust test guideline should facilitate the generation of highly reproducible, high-quality multi-*omics* data.

**Figure 1:**
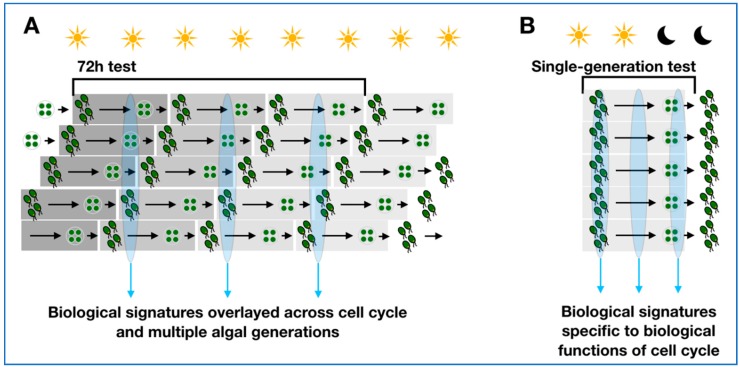
Abstract representation of the differences in sample composition between algal cell cultures grown in (**A**) continuous lighting over 72 h—as routinely used in OECD toxicity testing, and (**B**) alternating light:dark cycles for a single algal generation—as we propose and investigate in this study.

Current algal growth inhibition test guidelines [2,3] recommend culturing in constant lighting, a condition that induces rapid drift and loss of cell-cycle alignment over a population of algae that are responsive to light-regulation of the cell cycle [11]. In such a culturing regimen, samples for molecular analyses taken at any given time-point over the test duration will contain a cross-section of individual cells performing simultaneously all of the biological functions in the algal cell cycle, leading to averaged molecular signatures that are non-specific to biological functions or condition dependent perturbations (Figure 1A). Equally, apical endpoints (e.g., inhibition of cell division in algae) will be observable throughout this time course, preventing the possibility of biological and temporal anchoring of hypothesised mKEs to the adverse outcome (AO). To emphasize the challenging nature of interpreting molecular results from such experiments, an analogy is drawn to a hypothetical rodent bioassay with inclusion of ante-natal, neonatal, adolescent, middle aged, old aged and pregnant animal subjects, and pooling tissue samples from all of these life-stages to infer a chemical mode of action (MoA). There is a logical argument that just as life stage is controlled in vertebrate and invertebrate animal testing, similar considerations should be given to algal toxicity testing, which regrettably have so far been limited in scope [12,13].

Conventional algal growth inhibition tests commonly apply a 72 h exposure duration. Following introduction of cell cycle synchronisation, we aim to reduce the duration of exposure, and adjust patterns of sampling time-test duration for high-throughput screening, to ultimately enable discovery of mKEs occurring before (and that are predictive of) acute growth inhibition. Here, the prime consideration is the biological anchoring of the test duration to the life-cycle of a single algal generation (Figure 1B).

While *omics* technologies hold considerable promise for the discovery of MoA(s) [14,15,16,17], to date the majority of *omics* studies have been too small-scale to deliver on this promise in algae research. Seminal studies in microalgal toxico-*omics* include those by Nestler et al., Jamers et al. [18,19] and Pillai et al. [20]. However, the majority of past study designs either tended to (i) scrutinise only single snapshots of molecular perturbation [16,18,21,22,23,24,25], (ii) apply test durations which lack a biologically-justified anchoring of sampling time-points [19,26,27,28,29,30,31], and/or (iii) utilise non-synchronised cultures over time-course experiments [20,32,33,34,35]. Overall, new robust, miniaturised and scalable designs in routine algal toxicity testing are required [36]. However, large scale studies factoring time and toxicant exposure are currently not supported by conventional microalgal toxicity test practices. For instance, recent multi-*omics* studies on *C. reinhardtii* feature experimental designs that are restricted in sample size, analytical methodology, and time-points due to lack of biomass [37]. This challenge of generating sufficient biological material for *omics* analysis is particularly aggravated during the testing of volatile toxicants. Due to the CO_2_-dependence of natural photoautotrophic growth, existing volatile test systems are incompatible with above-mentioned experimental designs.

The overarching aim of the current study was to overcome the existing limitations in the algal growth inhibition test in the context of extending its applicability to *omics*-driven toxicological biomarker research. Following some initial modifications to the culture media, the first objective was to demonstrate the benefits of synchronised algae cultures for molecular studies using a combination of untargeted metabolomics, cell counting and flow cytometry. The second objective was to implement a highly replicable, closed-vial chemical exposure system with optimised *C. reinhardtii* growth at high inoculation cell densities, while minimising chemical volatilisation and test duration. The third objective was to demonstrate the application of untargeted metabolomics to this closed-vial test system by characterising the repeatability of the metabolic perturbations to a model narcotic, chlorobenzene, across three independent exposure experiments.

## 2. Results

### 2.1. Synchronised Versus Non-Synchronised Algae Cultures for Molecular Studies

**Figure 2:**
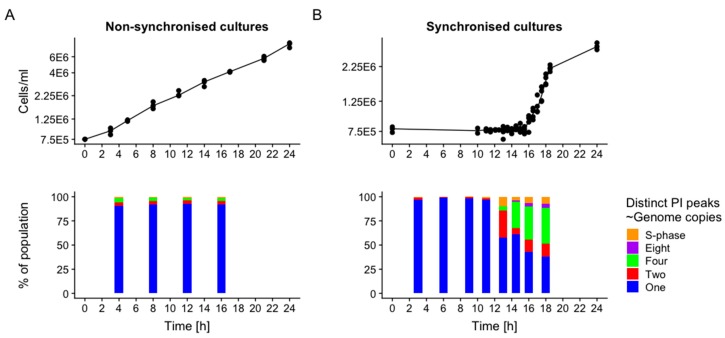
Cell density (**Top**) and number of distinct peaks in PI fluorescence intensity (**Bottom**; ~ amount of genomic material found in each zoosporangium or zoospore) indicating genomic division events during multiple-fission cycles for non-synchronised (**A**) or synchronised (**B**) cultures. For synchronised cultures, light:dark transition occurred at 12 h.

Cells grown in constant lighting or alternating 12 h:12 h light:dark conditions were compared regarding time-point specificity of zoospore release (i.e., increase in cell number and density) and mitotic activity, over a time-course of 24 h. A clear logarithmic increase of cell density over the whole measurement period (thus a constant presence of zoospore hatching events) were observed in the cell populations grown in constant light (Figure 2A). By contrast, for cell cultures grown in alternating 12 h:12 h light:dark conditions, an increase in cell number occurred exclusively after the light:dark transition in a narrow time window of approximately 2.5–3 h starting around the 16 h time-point, indicating a high population-wide coordination of zoospore release from zoosporangia within the experimental time-frame.

A similar, although phase-shifted, dynamic process was observed when mitotic activity between the two culturing regimens was compared with flow cytometric measurements (Figure 2). Cells were analysed for fluorescence of DNA-intercalating fluorophore propidium iodide after a nucleotide staining procedure and enzymatic RNA degradation. Cell cultures grown in constant lighting conditions comprised either single cell zoospores, zoosporangia enclosing two or four zoospores, or cell particles undergoing S-phase, to narrowly-defined percentages over the whole course of the experiment (single genome copy zoospores 92.07 ± 0.77% of population, two-genome copy zoosporangia 3.25 ± 0.26%, four copy 3.21 ± 0.77%, cells in S-phase 1.48 ± 0.26%). Cell cultures from alternating light:dark conditions were composed of highly variable and time-dependent cell population, dominated by cells containing a single genome copy (SGC zoospores) at all time-points before the light:dark transition at 12 h (97.06 ± 1.53%). Yet at the 13 h time-point, these SGC zoospores comprised only 58% of the population, with a concordant increase in cell particles in S-phase, or zoosporangia containing two or four genome copies. The number of cells containing ever greater numbers of genome copies grew exponentially at later time-points, concordant with the occurrence of multiple-fission cycles of the cells. Replication activity started as early as 11 h into the light-phase (cells in S-phase at 11 h significantly increased from 9 h, Welch’s t-test *p* = 2.7 × 10^−3^). Nuclear division events were observed at merely 1 h after the light:dark transition (13 h time-point). By comparison, zoospore hatching and respective increase in cell density was only observed after 15.5 h.

**Figure 3:**
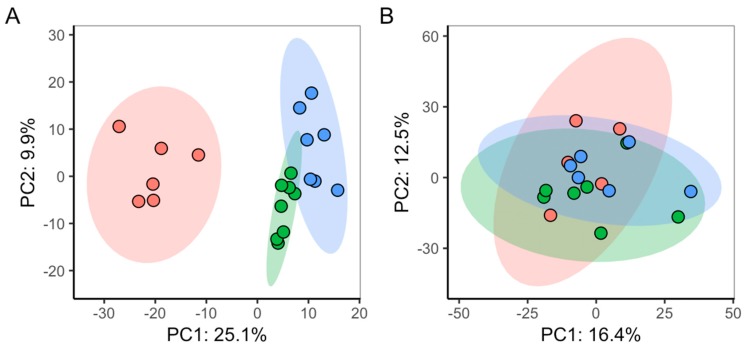
PCA scores plots comparing the DIMS metabolic phenotypes of (**A**) synchronised algal cell cultures grown in alternating light:dark conditions, and of (**B**) non-synchronised algal cell cultures grown in constant light. Plotted are PCA scores for samples taken at 4 h 15 min ±15 min (Red), 8 h 15 min ±15 min (Green)) and 12 h 15 min ±15 min (Blue).

To analyse the impact of light-induced cell cycle synchronisation on the metabolic phenotypes of algal cultures, direct infusion mass spectrometry (DIMS) based metabolomics was applied to cells grown in alternating light:dark conditions (synchronised cell cycles) versus grown in constant lighting (non-synchronised). Initially, two small optimisation studies were conducted; the first to determine the number of washes required during the extraction of metabolites from the algal cells; the second to optimise the dilution of the metabolite extract that was infused into the mass spectrometer (see Supp. Info SI-1 and SI-T1). Basal (unexposed) algal cultures were sampled from each culturing regimen at 4 h 15 min (±15 min), 8 h 15 min (±15 min) and 12 h 15 min (±15 min) post-seeding (*n* = 8 for each light regime and time-point). Intra-study quality control samples (QC) were derived from a single pool of aliquots from each biological sample. Using the established measure of median relative standard deviation (mRSD) of measured metabolite features as a descriptor of variance [38], technical variability over the dataset was estimated from QC samples [39], and total (dominated by biological) metabolic variability was assessed from groups of biological samples. mRSD of all *m*/*z* features for the QC samples in the analysis of the synchronised cell cultures was 9.1%, while QC mRSD for analysis of non-synchronised cell cultures was 12.1%. These results indicate low technical variability and hence high quality of the DIMS metabolomics data, as previously described by Parsons et al. [38]. Total mRSD for synchronised cultures was as follows: 25.1% (4 h 15 min ± 15 min), 27.4% (8 h 15 min ± 15 min) and 30.6% (12 h 15 min ± 15 min); while for non-synchronised cultures: 36.2% (4 h 15 min ±15 min), 27.6% (8 h 15 min ±15 min) and 33.8% (12 h 15 min ± 15 min). These values indicate a slightly higher biological variability in non-synchronised cultures *versus* synchronised cultures. Unsupervised multivariate analysis (principal components analysis, PCA) compared the metabolic phenotypes of the algae as a function of lighting regime and time-course (Figure 3). Outliers were removed if their measurements exceeded the 95% confidence interval derived for all the biological samples, within each dataset (4 samples in non-synchronised study, 2 samples in synchronised). No metabolic separation of time-points was observed for non-synchronised cell cultures grown in constant lighting conditions. However, for cells grown in alternating light:dark conditions, a progression of their metabolic phenotypes was discovered over time (along PC1). Statistical analysis of the PC scores for each sample indicated significant differences in PC1 (ANOVA, *p* = 1.8 × 10^−11^) and PC2 (*p* = 6.39 × 10^−3^) scores between metabolic phenotypes of the time-points for cells with synchronised cell-cycles, but not for non-synchronised cell cultures (ANOVA, PC1 *p* = 0.84, PC2 *p* = 0.85). This result indicates homogeneity of the metabolic composition of samples from non-synchronised cultures, and discrete compositions over time of those from synchronised cultures. Given the data, all subsequent studies were conducted using synchronised cell cultures.

### 2.2. Validation of C. reinhardtii Test System for Volatile Substances

To establish the validity of the medium and vial culturing system, growth rates of synchronised *C. reinhardtii* cultures and medium pH in capped-vials (7.5 × 10^5^ cells/mL inoculation density, 10% vial air space) were checked against OECD 201 Test Guideline criteria [3] prior to initiating dose-response experiments in the vials. Adequate growth rates of >0.92^−d^ (mean 1.14^−d^; *n* = 4) and a pH drift < 1.5 (mean 0.25; *n* = 4) over a 24 h incubation period were achieved, meeting the required specification (Supplementary Figure S2).

**Figure 4:**
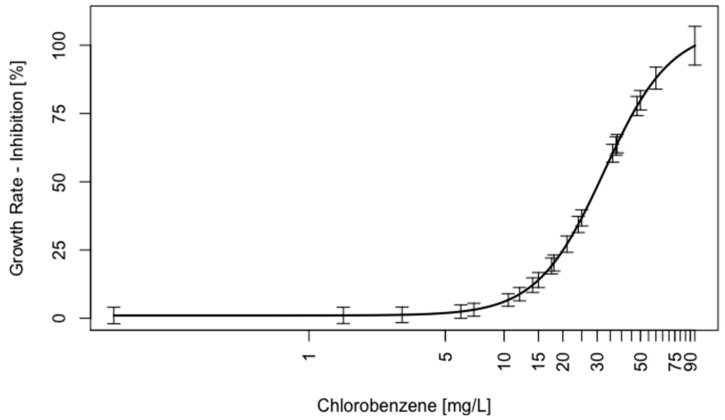
Dose-response curve (4 parameter log-logistic model) of 24 h algal growth inhibition experiments of chlorobenzene. The graph represents data from three independent experiments (*n* = 4 per concentration), error bars correspond to 95%-confidence intervals of fitted curve.

Subsequently, the modified capped-vial volatile test system was checked for robust and repeatable generation of concentration-response data, specifically for the traditional apical endpoint of algal growth, for both an OECD-recommended reference test substance 3,5-dichlorophenol (Supplementary Figure S3; [3]) and the volatile model toxicant chlorobenzene. The exposure period used was 24 h, aligning with both the cell culture studies presented above. An EC_50_ = 0.93 ± 0.09 mg/L was derived for 3,5-dichlorophenol, while growth data from three independent chlorobenzene exposure experiments yielded an effective concentration estimate of EC_50_ = 32.5 ± 3.6 mg/L (Figure 4). Of particular note is the high repeatability (low variation) in the growth data derived from the synchronised culture test system.

**Figure 5:**
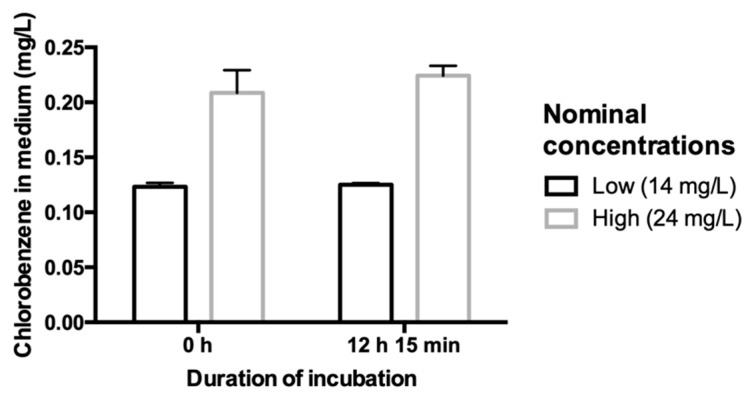
Barplots comparing chlorobenzene concentrations in test system medium, measured using GC-MS, to assess whether any loss occurs over the test duration. At 0 h and after a 12 h 15 min exposure period, two concentration groups ‘Low’ (nominal 14 mg/L) and ‘High’ (nominal 24 mg/L) test groups were measured. Bars represent mean ±1sd (*n* = 4 per group).

To assess the suitability of the modified capped-vial exposure system for maintaining stable concentrations of a volatile toxicant in the medium, over the test duration, extra vials were added to the experimental design and GC-MS analysis conducted on chlorobenzene levels in the medium of these sacrificial vials sampled at the beginning (0 h) and after 12 h 15 min incubation. Exposures were conducted and samples taken at each time-point for both low (14 mg/L) and high (24 mg/L) nominal chlorobenzene concentrations (*n* = 4 for each time-point and concentration; Figure 5). Concentrations of chlorobenzene from medium sampled at 0 h were slightly lower than nominal (low group: 12.32 ± 0.31 mg/L; high group 20.87 ± 1.78 mg/L). However, they remained stable until 12 h 15 min (Low: 12.51 ± 0.13 mg/L, High: 22.43 ± 0.78 mg/L). No significant differences were observed between the 0 h and 12 h 15 min medium chlorobenzene concentrations for both levels (Welch’s t-test, Low *p* = 0.39, High *p* = 0.24).

### 2.3. Repeatability of C. reinhardtii Metabolic Phenotypes in Test System

To characterise the reproducibility of the metabolite phenotypes of synchronised *C. reinhardtii* cultures grown in the capped-vial exposure system, DIMS metabolomics was conducted on samples generated over three independent experiments. Each biological batch comprised of highly replicated algal cultures in pure growth medium (control, *n* = 10) and cultures exposed to 25 mg/L chlorobenzene (CB, *n* = 10), with all samples harvested after a 3 h incubation. This exposure concentration was selected based on the previous dose-response study (Figure 4) with the 3 h time-point assumed to capture early metabolic perturbations induced by the narcotic substance; exposures were conducted early in the light phase of the 12 h:12 h light:dark cycle. Algal cell samples of each biological batch were stored at −80 °C, then all samples were extracted and DIMS metabolomics analysis (positive ion mode, polar metabolite fraction) performed as a single analytical batch. This design allowed biological variation between the exposure experiments to be isolated and identified.

First, RSD and median RSD values were calculated for all batches and groups to characterise technical and total (dominated by biological) metabolic variability. mRSD of all *m*/*z* features within intra-study QC samples was 9.3%, confirming low technical variability and high quality of the instrumental analysis (Supplementary Figure S4). RSD distributions and mRSD values suggested observable, although small, differences between biological batches, with batches 2 and 3 displaying lower mRSD than batch 1. mRSD values in biological batches 1, 2 and 3 were—for Control groups—26.0%, 23.1% and 22.4%, and in CB groups 26.7%, 24.1% and 24.1%.

**Figure 6:**
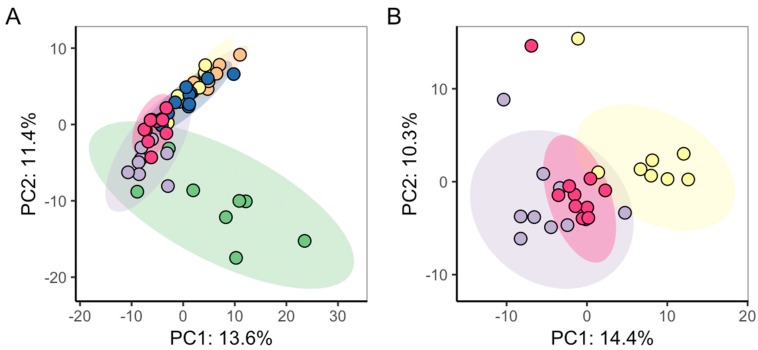
PCA scores plots (PC1 vs. PC2) visualising (**A**) the metabolic differences across three exposure studies (termed biological batches), with each batch comprising of control (*n* = 10) and CB-exposed (*n* = 10) samples; (**B**) only the control samples from each batch. Sample groups are biological batch 1 Control (Purple) and CB (Green), batch 2 Control (Yellow) and CB (Orange), and batch 3 Control (Red) and CB (Blue).

PCA was applied to study the metabolic variability inherent to the complete dataset. Two outliers were removed (batch 1, CB samples) as they exceeded the 95% confidence interval of the complete dataset, and PCA conducted on the 56 remaining samples. The PCA scores plot (Figure 6A) revealed that the three batches were not identical, with batch 1 CB samples showing the highest intra-group variance as well as differing from the batch 2 and 3 CB samples in multivariate space. Additionally, while the metabolic effects of CB relative to the control samples were apparent for batches 1 and 3, a minimal difference between controls and CB-treated samples was observed for batch 2.

To further investigate, PCA was conducted on the control samples alone, from all three biological batches (Figure 6B and Supplementary Figure S5), to determine the extent to which the baseline algal metabolome varied across studies. ANOVA of the PC scores for the three control groups indicated significant differences along PC1 and PC3 (*p* = 7.15 × 10^−6^ and 3.84 × 10^−4^, respectively; Table 1), indicating certain inter-batch metabolic variation between the three biological batches. Similarly, PCA and subsequent ANOVA of PC scores were applied to just the CB samples, which also revealed significance of metabolic differences between the three biological batches (Supplementary Figure S5 and Table 1). Potential sources of this batch effect included metabolic variation in the cultures themselves (over the duration of the three studies), the sampling of the algae, and/or other operator effects.

**Figure 7:**
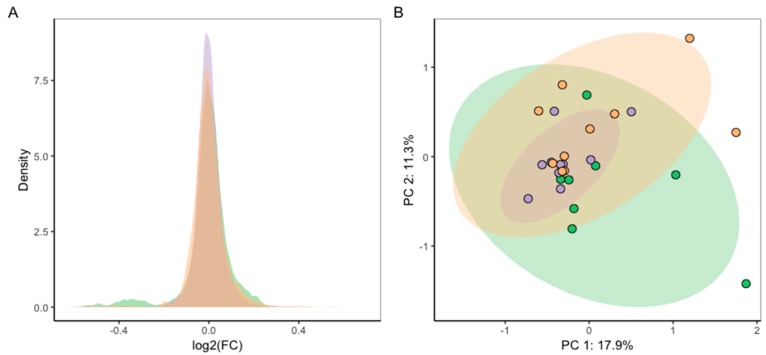
To increase comparability of exposure effects over the three repeated experiments, *m*/*z* feature intensities in CB groups were normalised to their batch-respective control groups by calculating the log_2_ fold-change of *m*/*z* features: (**A**) density distribution of LFC values for each batch (log_2_ (median[Int_CB_]/median[Int_Control_]), per *m*/*z* feature); (**B**) PCA scores plot from analysis of LFC values of all three batches (log_2_ (Int_CB_/median[Int_Control_]), for each *m*/*z* feature and for each of 10 CB samples).

To achieve the high level of repeatability required for toxicity testing, we explored the effect of normalising each batch of exposure data to the corresponding control metabolic phenotypes. First, we calculated the log_2_ fold-change (LFC) for every *m*/*z* feature (peak), specifically by determining log_2_ of the ratio of the median feature intensity in the CB group over the median intensity in the respective control group (log_2_ (median[Int_CB_]/median[Int_Control_]), for each *m*/*z* feature). This calculation was repeated for each of the three batches. The resulting density distributions of LFC values, one for each biological batch, were visually similar (Figure 7A). Yet upon statistical testing, these LFC batch values were significantly different (Kruskal-Wallis test, *p* = 1.38 × 10^−12^).

To further explore the effect of normalising each batch of exposure data to the corresponding control metabolic phenotypes, specifically using PCA, log_2_ fold-changes were recalculated individually for the metabolite features of each of the *n* = 10 samples in the CB group relative to the median feature intensity of their respective control group (log_2_ (Int_CB_/median[Int_Control_])). This allowed PCA of these LFC values to reveal any metabolic differences between the effects of CB-treatment normalised to each batch-control, for all three batches (Figure 7B), with the scores plot now highlighting the significant overlap and therefore consistency of the batches. Indeed, ANOVA indicated no significant differences between PC1 and PC3 scores (PC1 *p* = 0.20, PC3 *p* = 0.11). However, slight batch differences were still detectable along PC2 (*p* = 0.015). Importantly, initial batch effects in PCA (Figure 6A,B) were greatly decreased by applying this normalisation strategy to the relevant batch-controls (Figure 7B).

We next evaluated the repeatability of the CB-induced metabolic perturbations in the three exposure experiments from the context of molecular biomarker discovery. This was achieved by applying set enrichment analysis [40] to the metabolomics data. This statistical process of comparing the rank order of genes, based on the magnitude or significance of their responses to multiple experimental conditions, was here applied on the rank order of mass over charge (*m/z)* metabolic features. Three comparisons were made (Table 2).

First, the top 100 LFC *m*/*z* features from batch 1 were designated as metabolite set (termed set batch 1, or S_B1_), and used to interrogate the rank order of *m*/*z* metabolic features obtained from the other two batches. The significance of this set enriching the leading edges (LE) of each of the two batches was calculated using the normalized enrichment score (NES) and by estimating a false discovery rate (FDR). This enrichment test was twice repeated, for S_B2_ and S_B3_. The results indicate that top 100 LFC *m*/*z* features for from each batch are strongly and significantly enriching all biological batch-metabolite set combinations (Table 2), concluding that a common and consistent subset of metabolic markers as a toxicological signal was discovered across all the biological batches, despite small batch effects in PCA.

**Figure 8:**
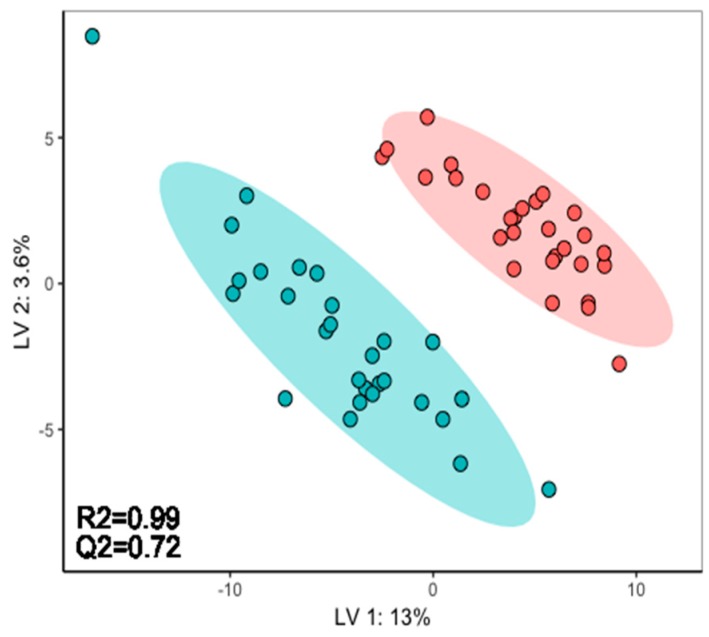
PLS-DA scores plot visualising the metabolic effects in *C. reinhardtii* following exposure to 25 mg/L chlorobenzene (red circles) relative to unexposed controls (green circles). All samples from three independent exposure experiments (biological batches) were pooled in this analysis.

Finally, we sought to evaluate the metabolic perturbations in the three exposure experiments using a supervised multivariate analysis, which focuses on the most consistent changes induced in the CB-treated samples. Specifically, partial least squares-discriminant analysis (PLS-DA) was conducted on *m*/*z* features of all of the samples from the three biological batches. The algorithm selected an optimum of 5 latent variables for the classification model. While the control and CB samples were clearly separated in the PLS-DA scores plot of latent variable 1 vs. 2 (Figure 8), a robust interpretation of these results required any over-fitting of the PLS model to be assessed. Following 10-fold internal cross-validation, the PLS model yielded an R^2^ = 0.99 (amount of variation in data explained by model) and Q^2^ = 0.72 (goodness of fit), which indicated good generalisability.

## 3. Discussion

Chemical risk assessment by new approach methodologies requires test protocols with experimental designs that are tailored to applying *omics*, so to gain insights into toxicity mechanisms on a global molecular level and for discovering mKEs [6,15,36,41]. We here developed an extended variant of the regulatory algal growth inhibition test towards routine large-scale investigation of molecular toxicological processes. We achieved this by combining population-wide synchronisation of molecular functions in algal cell cultures with an easily scalable and abbreviated single-generation test system, suitable for testing of both soluble and volatile toxicants, and characterised the quality of generated molecular data using metabolomics.

### 3.1. Synchronised Cultures in Algal Toxicity Testing

For various unicellular green algae commonly applied in growth inhibition tests (e.g., genera *Chlamydomonas, Raphidocelis*, *Desmodesmus*, *Scenedesmus*, *Chlorella*), the phenomenon of light-driven cell cycle synchronisation is a known manifestation of evolutionary adaptation to scarcity of light in natural environments that can be experimentally induced by introduction of alternating light:dark cycles during incubation [42,43,44]. The degree of induced coordination between cells is close to absolute [45], nonetheless the benefits of this algae culture synchronisation to the context of environmental toxicology have previously only been given brief consideration [12]. Exposure and effect analysis of multiple life-stages within the same experiment are commonly avoided in toxicity assessments due to introduction of various confounding factors [46]. Initial matching of test organisms by life-stage is prescribed in the standardised OECD test criteria for most environmental model organisms, such as water flea, birds, bee, annelids, springtail, molluscs, amphibia and fish [47]. As one of the few cell-based in vivo systems, microalgal bioassays do not feature the requirement of life-stage/cell-cycle matching [2,3]. Commonly prescribed test conditions, including constant lighting, may further be considered non-representative of natural environments, and drives suboptimal metabolic efficiency and rapid cell cycle misalignment [11,48,49].

In the presented data, coordination of genomic division and sporulation events over the complete algal population in cultures grown in light:dark cycles, and lack thereof in cultures grown in constant light, was confirmed via electronic cell counting and flow-cytometry. This difference was indicative of cell cycle synchronisation in algae grown under alternating light cycles [45]. From the perspective of mechanistic (eco-)toxicology, one significant benefit of the observed population-wide coordination of nuclear division and sporulation is the ensuing occurrence and measurability of the algal adverse phenotypic endpoint within a narrow time-window across the cell population. This in turn enables temporal phenotypic anchoring of the AO to prior molecular perturbations, thereby defining the AOPs [9]. In contrast, described coordination and opportunity of mechanistic anchoring is lost in algal cell culturing using constant light regimes [11], due to consistent occurrences of nuclear division and sporulation over the entire experimental time-frame.

In mammalian cell models, synchronisation is routinely applied to enable detailed investigations into specific phases of the cell cycle [50]. In microalgae such as *C. reinhardtii*, synchronisation of cell cycle was applied to study various biological processes [51,52,53,54,55], however its explicit importance for toxicological investigations remains undervalued [12,13]. Our experiments comparing the metabolite phenotypes from *C. reinhardtii* grown under the two culturing regimens emphasised substantial and significant changes in the molecular composition in the synchronised *C. reinhardtii* cultures through time. Cells are tightly aligned in their progress through the entire cell cycle up to nuclear and cellular division [56] and characteristically evolving metabolic and transcriptomic patterns have previously been linked to this progression in *C. reinhardtii* [51,57]. In both pro- and eukaryotic cells, evidence is mounting that metabolic activity is not merely affected by position in the cell cycle, but a major driver of it [58,59]. Accordingly, distinct changes of metabolic patterns along the cell cycle were discernable in the scope of metabolomics analysis of synchronised cell populations. However, a complete lack of such an effect was apparent in cultures grown in constant light as prescribed by regulatory guidelines. Under non-nutrient-limited exponential growth conditions, non-synchronised cultures grown in constant light will comprise algal cells from every possible stage of the cell cycle, irrespective of experimental time-point. Although individual cells still progress through cell cycle, extracted samples for molecular studies comprise cross-sections over the whole complement of cell cycle-dependent biological functions, as evidenced by the high similarity of metabolic profiles between successive time-points over the experimental time-frame.

Metabolic phenotypes from samples of synchronised algae cultures taken at specific time-points during the exposure period were representative of narrow windows of biological functions anchored to cell cycle. The equivalent is true for perturbations of cellular functions (mKEs), which can be precisely measured over respective time-course analyses. Light:dark cycle-induced algal cell cycle synchronisation should thus be able improve *omics*-driven time-course analysis of mKEs by avoiding phase-shifts of molecular signals in the test system [60,61]. In non-synchronised cultures, discrete sampling intervals cannot be precisely rooted to cell-cycle-dependent biological processes. Interpretation of toxicological mechanisms remains limited to snapshots of overlaying molecular cross-sections of algal populations, and molecular phenotypes are averaged over the heterogeneous cell population within a sampling time-point. It is concluded that synchronised cell cultures in this context represent an approach to study molecular biological processes and their perturbations with strongly reduced variability, enabling increased analytical resolution of biological processes and their perturbances.

### 3.2. Design and Validation of an Alternative Volatile Testing System

Reducing the duration of a conventional algal test protocol would increase sample throughput and thus facilitate applicability for high throughput *omics* technologies, e.g., *for* applications in read-across and prioritisation. For the purpose of discovering mKEs, the commonly prescribed 72 h test duration represents an arbitrarily-selected timeframe to capture low-concentration delayed-onset mechanisms of toxicity. A warranted question in this context is why the standard test duration should not be increased to even longer durations to optimise sensitivity of the system to lower concentration effects (bioaccumulation or damage). Given the selection of inhibition of cellular division as the AO for microalgal toxicity, it can be assumed that AO-inducing key events must occur and be measurable within perturbations of preceding biological functions [62,63] and thus within previous cell cycle stages. Justification of the test duration in microalgal exposure studies aiming to discover mKE and infer chemical MoA, therefore, should not hinge upon absolute length, but on biological anchoring, i.e., the minimal time required to link molecular stress responses to successive phenotypic effects. The reduction of test duration encompassing a single generation of synchronised cells (Figure 1B) is proposed in this context. It may be argued that an optimal sampling strategy would extend exposure and sampling to the respective dark phase of the alternating light:dark cycle after sporulation, allowing the effects of a chemical on dark cycle processes to be examined. The practical implementation of such a protocol is not readily feasible, risking activation of biological functions in algae exposed even to minimal light indicative of a commencing cell growth cycle [51,64]. Furthermore, algal biological activity in complete darkness is indicated to be largely limited to mitochondrial energy metabolism, which is maintained throughout the light phase [51,65,66,67]. Consequently, the proposed time-frame from commencement of light phase to sporulation covers the single largest set of molecular and cellular functions within a cell cycle that chemicals may perturb.

Algal toxicity testing of volatile chemicals has historically proven problematic when combined with requirements for test miniaturisation and increased harvestable biomass, due to natural autotrophic requirements. Conventional agitation- and aeration-based exposure vessel systems become highly cumbersome to maintain with increasing experimental scale, and the stability of mid-concentration exposures is difficult in open flask culturing. A variety of solutions has been proposed to address the issue, including tailored complex flasks [68,69,70] and variants of bottle or tube testing [71,72,73,74,75,76,77,78,79]. The lower limit of *C. reinhardtii* biomass to achieve reproducible data in metabolomics and lipidomics studies has been reported at ca. 2–2.5 × 10^6^ cells per sample [80,81], and similar or higher for transcriptomics [51,82,83,84] or proteomics [22,85]. This precludes the application of existing methods to large-scale (multi-)*omics* studies due to low cell densities, problematic physical scalability, disturbance of phase equilibria during repeat-sampling procedures, strong pH drift, or a combination thereof.

Here we developed and validated a hybrid sacrificial capped-vial exposure system to address existing shortcomings. The achieved biomass of >7.5 × 10^6^ harvestable cells per test vial has been substantially increased from comparable vial systems [75,76,77] and is sufficient to generate biological material for the demands of multiple *omics* technologies in parallel. In the exposure system, synchronous *C. reinhardtii* cultures performed well with mean growth rates at 1.14^−d^, well above the validity requirements of 0.92^−d^ [3]. Low pH drift of 0.25 over the test duration was achieved (required <1.5; [3], precluding risk of changing bioavailability due to photosynthesis-driven pH changes, an issue more likely observed in other volatile test systems [68,71,86].

Past studies to hypothesise on chemical MoA in algae were frequently limited in exploring the temporal dynamics of toxicity. Many existing multi-*omics* studies attempting to deduce a MoA of chemicals as a dynamic processes in *C. reinhardtii* have employed experimental designs with relatively low numbers of sampling time-points [18,19,21,22,23,24,37,87]. Again, the required experimental designs are not sufficiently supported by conventional microalgal toxicity test practices, and the scarcity of existing publications with time-series analyses of algae is partly due to a relative lack of physical scalability. The volatile testing system reported here represents a miniaturised and abbreviated variant of the conventional algal growth inhibition test, reorienting its focus on molecular events predictive of a measurable acute adverse outcome after a single clonal generation of *C. reinhardtii.* By utilising small glass vials, parallelisability of test vessels and thus factorisation of experimental variables can in principle be drastically increased in individual experiments. Capacity to test hundreds of vial replicates in parallel, per individual toxicity test, has been achieved in multiple other studies in the author’s laboratory (in prep.), enabling experimental designs to accommodate for high replication, temporal resolution and multiple exposure concentrations.

To demonstrate the generation of reproducible results by the designed testing approach for soluble and volatile toxicants, dose-response experiments of 3,5-DCP and chlorobenzene were performed to compare generated data to existing knowledge. EC_50_ value for the reference substance 3,5-DCP was estimated at 0.93 ±0.09 mg/L, well within the range of 0.5 to 6.1 mg/L (48 to 72 h-EC_50_) observed for *C. reinhardtii* and other green algae [88]. The determined 24 h-EC_50_ of the highly volatile chemical chlorobenzene for *C. reinhardtii* was estimated at 32.52 ±3.64 mg/L (data of three repeats), again within range of published closed-test system data (*P. subcapitata* 48 h-EC_50_ = 14.4 mg/L [79]; *Selenastrum capricornutum* 96 h-EC_50_ = 12.5 mg/L, [69]), and drastically reduced compared to results derived from open-flask testing (*S. capricornutum* 96 h-EC_50_ = 224 mg/L [89]). When accounting for inter-species variability in sensitivity and differences in test exposure durations and physical setups [69], the estimated EC_50_ suggests adequate performance of the vial exposure system within expected ranges of variation.

Another requirement of the exposure system was to enable stable exposure concentrations of volatile toxicants over the experimental timeframe. Measurements of chlorobenzene levels at the start of the experiment were lower than nominal (14 mg/L nominal measured at 12.32 ± 0.31 mg/L; 24 mg/L nominal measured at 20.87 ± 1.78 mg/L), however the levels did not change significantly over the 12 h 15 min incubation period. This indicates chemical loss during initial stock preparations, however high stability of concentration levels over the incubation period. According to Henry’s law constant, fraction of chlorobenzene in the gas phase is calculated as %CB_g_ = V_g_/(V_g_ + (V_L_·R·T/K_H_)) with V_g_ = 10% vial gas phase, V_L_ = 90% vial liquid phase, T = 298.15 K, R = 8.205 ± 10^−5^ m^3^·atm/(K·mol), K_H_ = 3.7 × 10^−3^ atm·m^3^/mol [90]. At equilibrium, only 1.65% of chlorobenzene theoretically partitions into the gas phase. Similar calculation for the expected partitioning of 204 Volatile Organic Compounds [91] suggests that 85% would partition less than 6% into the gas phase of the proposed exposure system. Concluding, stable exposure over the time-course of the experiment was well achievable using the designed capped-vial testing system, and minimal partitioning into the gas phase occurs during the experimental timeframe. Anticipation of and correction for initial volatilisation loss during stock preparation of highly volatile chemicals at inoculation is advised for further studies.

### 3.3. Characterising Variability and Repeatability of Metabolomics Data

Robust and reproducible determination of biomarkers and toxicological key events predictive of chemical insult from the algal metabolome is paramount for downstream utilisation of the generated data to support environmental hazard assessments. To characterise the reproducibility of data gained from our exposure approach, differences in the metabolite phenotypes between three repeated exposure studies were compared. Ranges of recorded median RSD values (20.86% to 24.99%) lay within the expected ranges for test organisms and cells, e.g., median RSD values have been reported for human cell lines between 20–22% [38].

Small batch effects could be observed between the baseline metabolic phenotypes across the three repeats of the exposure experiment when compared in PCA. A number of possible factors might have contributed to these differences, including subtle changes in laboratory maintenance that unknowingly affected the growth and biological activity of the sensitive algal cultures, as reported previously for microalgal bioassays [92,93,94,95]. We demonstrated that normalisation of the observed differences in algal stress response, by calculating log_2_ fold-change of the measured *m*/*z* feature intensities to their batch-specific biological baseline (controls), proved to mitigate existing batch differences.

Despite these small batch effects in PCA, the algal metabolic stress response appeared to be regulated by a consistent mechanism across studies, as indicated by PLS-DA on pooled data. Additionally, we applied set enrichment analysis to metabolomics data, an approach originally developed for analysing gene set enrichment [40] which lately has become increasingly applied in metabolomics [96,97,98,99]. Set enrichment analysis was conducted to characterise the overall degree of similarity of whole sets of metabolic markers associated with chlorobenzene exposure between the repeat experiments. Metabolite sets containing the most important class markers within each of the biological batches were found to be significantly and strongly enriched within the top ranks of *m*/*z* features within the other batches, adding further support to our conclusion that a consistent toxicological effect could be measured.

## 4. Materials and Methods

### 4.1. Algae Culturing

Axenic *Chlamydomonas reinhardtii* Dangeard (1888) CC-125 wild type mt+ was purchased from the Culture Collection of Algae and Protozoa (CCAP, Dunbeg, UK). The strain was maintained heterotrophically on agar plates (1.5% *w*/*v* agar in tris-acetate-phosphate media) on laboratory shelves at 23 °C until liquid inoculation. A modified growth medium (termed *Chlamydomonas* growth medium; CGM) was developed here based on Sueoka’s high salt medium supplemented with Kropat’s trace elements [100,101] with the following modifications. The final concentrations of the divalent inorganic salts CaCl_2_*2H_2_O and MgSO_4_*7H_2_O were increased to 50 mg/L and 100 mg/L, and the concentration of total phosphates was reduced by 92.5% to 54 mg/L KH_2_PO_4_ and 108 mg/L K_2_HPO_4_ to decrease both palmelloid formation and mass spectrometric ion suppression due to phosphate accumulation in *C. reinhardtii* cells [102,103,104]. To increase inorganic carbon levels, the growth medium was supplemented with 500 mg/L Na-HCO_3_. As *C. reinhardtii* growth is optimal between pH 5.5 to 9 [105,106], pH was adjusted to 6 to shift carbon equilibrium towards photosynthetically-available carbonic acid species [107]. To retain buffer capacity for a photosynthesis-driven change in medium pH, CGM was supplemented with 20 mM MOPS buffer, similar to Renberg et al. [102].

The growth medium was sterilised by 0.22 µm-filtration to avoid degradation of MOPS, salt precipitation and shift of the carbon equilibrium caused by autoclaving [108]. For initial liquid stock cultures, agar cultures were inoculated into 35 mL CGM within foam-bunged 250 mL wide-neck glass flasks. Cultures were incubated in a Multitron Pro rotary shaking incubator (Infors HT, Bottmingen-Basel, Switzerland) at 25 °C and 200 rpm orbital shaking. Lighting conditions were either set as constant lighting (effecting non-synchronised cell cycles), or as a 12 h:12 h light:dark cycle (inducing population-wide synchronization of cell cycle). Light colour temperature was 8500 K. Algae were adapted to this lighting and autotrophic growth conditions in liquid cultures for at least 120 h prior to experiments.

The first study compared the metabolic phenotypes and mitotic activity of cell cultures grown under continuous lighting versus those grown under alternating 12 h:12 h light:dark cycles. Algae grown in either condition were seeded in vials as described below. Samples were taken at 4 h 15 min (±15 min), 8 h 15 min (±15 min) and 12 h 15 min (±15 min) post-seeding. 5.25 × 10^6^ (7 mL) were taken from synchronised samples at every time-point, and sampling volume of the non-synchronised samples were adjusted to match this cell number by prior electronic cell counting. The latter was performed using CASY TT cell counting technology (Roche Innovatis AG, Basel, Switzerland) which operates via electric pulse area analysis to count suspended particle. As described below, cells were harvested and stored at −80 °C until combined metabolite extractions.

Over the course of this study, further samples were taken for flow cytometric measurements of genomic material in cell particles at 4 h, 8 h, 12 h and 16 h post-seeding for non-synchronised cultures, and 3 h, 6 h, 9 h, 11 h, 13 h, 14.5 h, 16 h, 18 h post-seeding for synchronised cultures. 1 × 10^6^ cells were sampled (*n* = 3), centrifuged at 7000× *g* for 3 min (23 °C), pellets washed once in 1 mL phosphate-buffered saline (PBS), and cells fixed in 70/30 ethanol/water (both HPLC-grade, Fisher Scientific, Loughborough, UK) for 24 h at 4 °C. This last step served to permeabilise cells for propidium iodide (PI) and to extract chlorophylls which might interfere with the PI emission spectrum [109] The cells were washed again in 1 mL PBS and incubated in the dark for 30 min in 500 µL PBS containing 0.1 mg/mL RNAse-A (Qiagen, Hilden, Germany) and 0.01 mg/mL PI in water (94%, Sigma-Aldrich, Gillingham, UK). For 1 × 10^4^ particles per sample, PI fluorescence was measured using a Becton Dickinson FACS-Calibur (488 nm excitation, bandpass filter 610/10), and analysed using open-source Flowing v2.5.1 (flowingsoftware.btk.fi). Absence of chlorophyll interference was confirmed with non-stained samples. Due to haploidity of *C. reinhardtii* vegetative cells, discrete peaks in per-particle fluorescence intensity histograms from cells sampled at a given time were equated either to the number of genome copies present within each cell particle (zoosporangia or zoospores) post nuclear division. Fluorescence areas in between discrete peaks were assigned as cells currently undergoing DNA replication and assigned as ‘S-phase’. Growth curves of both culture regimen were determined using CASY cell-counting, at 2–4 h intervals for non-synchronised cultures and at higher resolution (30 min intervals) at the light:dark transition for synchronised cultures.

For each sampling time-point, vials were of sacrificial nature to maintain carbon equilibrium. Synchronised cell culturing was determined as preferable to non-synchronised cultures, as described in results, and used in all further studies.

### 4.2. Chlorobenzene Exposures

**Figure 9:**
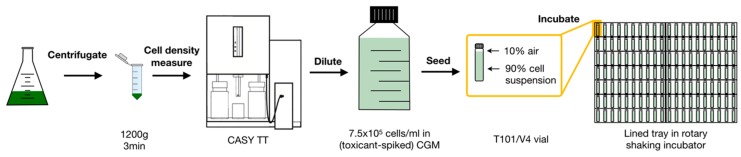
Individual steps for centrifugal concentration, cell density measurement, dilution, seeding of *C. reinhardtii* cell cultures into T101/V4 vials, and incubation of vials on a lined tray. Biological replicates in vials (*n* = 4 per experimental group) were generated from independent flask cultures.

To expose cells, quadruplicate independent culture batches of synchronised cultures were grown in open flask culture, and at commencement of the light phase the cell cultures were concentrated individually by centrifugal separation in 50 mL tubes at 1200× *g* for 3 min (Eppendorf 5920 r, Eppendorf, Stevenage, UK). The supernatant was decanted, cells were diluted in toxicant-spiked CGM to yield a final cell concentration of 7.5 × 10^5^ cells/mL, and seeded in clear 10.5 mL glass vials with aluminium cap inlay (N° T101/V4, Scientific Glass Laboratories, Stoke-On-Trent, UK) to establish 10% total gas phase volume within each vial (Figure 9). Vials were capped and incubated for 24 h, rolling free on a lined tray in the rotary incubator to maintain cell suspension. Preparation steps were restricted to a maximum duration of 30 min within the onset of light phase. Culture cell density for inoculation was scrupulously controlled and growth rates measured in technical duplicate using CASY cell counting.

A study conducting toxicity tests on synchronised cell cultures was performed using chlorobenzene (CB; 99.9%, Sigma-Aldrich, Gillingham, UK) as a volatile substance and 3,5-dichlorophenol (3,5-DCP; 97%, Sigma-Aldrich, Gillingham, UK) as the reference substance. All tests were performed with biological quadruplicates. *C. reinhardtii* was exposed to CB in three independent experiments at individual concentration ranges: 0, 15, 24, 38, 60, 95 mg/L (repeat 1, for range-finding); 0, 3.5, 7, 10.5, 14, 17.5, 21, 25, 37.5, 50 mg/L (repeat 2, adjusted range to encompass curve slope); 0, 1.5, 3, 6, 12, 18, 25, 36, 48 mg/L (repeat 3, for focus on environmentally-relevant lower end of dose-response curve). Cells were exposed to 3,5-DCP at 0, 0.6, 1.2, 2.4, 4.8 and 9.6 mg/L. A 4-parameter log-logistic dose-response model and effective concentration (EC) estimation was performed using [110] and drc package.

A further exposure study was conducted to compare the metabolite phenotypes of synchronised cell cultures in pure growth medium (control) versus cells exposed to 25 mg/L CB, repeated three times, to characterise the metabolic variability and repeatability of data generated in the exposure vial system. Ten biological replicates for each of the control and CB groups were seeded in capped vials, incubated for 3 h, harvested as described below, and cell pellets stored at −80 °C until metabolite extraction. This experiment was repeated three times in identical set-up with a duration of 5d between experiments. Metabolite extraction was performed for all samples of the three repeats combined.

### 4.3. Sampling and Metabolite Extraction for DIMS Metabolomics

#### 4.3.1. Sampling Procedure

Modifying the procedure by Lee and Fien [80], algae cell suspensions were quenched by injection into an equivalent volume of −78 °C 70:30 methanol:water (HPLC-grade, Fisher Scientific, Loughborough, UK) solution in 15 mL tubes over dry ice. Quenched cell suspensions were centrifuged at 3800× *g* and −11 °C for 3 min, and the supernatant aspirated. The number of washes was first optimised in a pilot study by comparing 10 samples from each of three groups: no wash, single wash of cell pellet, or two washes. Each wash comprised of resuspension of the cell pellet in 10 mL of −20 °C 35% methanol:water, then repeating the centrifugation and supernatant aspiration (described above). Between centrifugation and aspiration steps, samples were stored in a custom-manufactured mobile freezer unit between −20 °C pre-cooled aluminum beads to minimise metabolic activity and avoid freezing of suspension and thus risk of cell lysis at lower temperatures. After aspiration, pellets were flash-frozen in liquid nitrogen, and all samples stored at −80 °C until metabolite extraction. A single pellet wash was determined as optimal (see results in Supplementary Figure S1) and used for all further experiments in this study.

#### 4.3.2. Metabolite Extractions

Biphasic metabolite extractions were performed with a final solvent ratio of methanol:chloroform:water (chloroform HPLC-grade, Fisher Scientific, Loughborough, UK) of 2:2:1.8 [111]. Cell pellets were thawed in randomised subsets of 24 tubes on ice. Cell pellets were resuspended in 970 µL ice-cold 70:30 methanol:water, transferred into homogenisation tubes with 0.5 mm glass beads (VK05, Stretton Scientific, Stretton, UK) on ice, and immediately homogenised at 6800 rpm for 2 × 15 sec bursts using a Precellys-24 tissue homogeniser (Bertin Instruments). Samples were placed back on ice for 2 min, then the supernatant (742 µL) was transferred to a 1.8 mL glass vial (aluminum-lined caps) containing 795 µL of ice-cold 1:2 water:chloroform, to yield the final solvent ratio of 2:2:1.8. Vials were vortexed for 30 sec and centrifuged for 15 min at 2585× *g* at 4 °C to achieve phase separation. Half the polar phase was aliquoted to a 2 mL Eppendorf tube using a glass syringe for untargeted metabolomics of the polar metabolite fraction (the other half retained for a second metabolomics assay, i.e., a different ion mode mass spectrometry). Each aliquot was dried in a SPD11V SpeedVac sample concentrator (Thermo Scientific, Rugby, UK) at room temperature. Extracts were stored at −80 °C until reconstitution in injection solvent (below). The same procedure was performed to generate an extraction blank sample.

### 4.4. Direct Infusion Mass-Spectrometry (DIMS) Metabolomics

Sample preparation, direct infusion mass spectrometric analyses and data processing were performed after the procedure of Southam et al. [39] To maximise the number of reproducibly detected *m*/*z* features, the dilution of the reconstituted algal extracts was first optimised (see results in Supplementary Table S1). A reconstitution volume of 50 µL injection solvent (80:20 methanol:water containing 0.25% *v*/*v* formic acid (98%, VWR)) was found to result in the maximal number of unique *m*/*z* features post-processing, therefore this option was used for preparation of all further samples. Ten µL of each reconstituted sample was pooled into an intrastudy quality control (QC) sample. All samples (biological, extraction blank, intrastudy QC) were analysed as technical quadruplicates (10 µL) on an Orbitrap Elite mass spectrometer (Thermo Scientific) with direct infusion nanoelectrospray ionisation (nESI) source (Triversa, Advion Bisociences, Ithaca, NY, USA) in positive ionisation mode. Individual SIM-windows of samples were stitched into single mass spectra, ≥75% replicate filtered, global peak aligned with a 3 ppm error tolerance, extraction blank filtered, QC-based locally-estimated scatterplot smoothing (LOESS) signal corrected [112], and ≥80% QC sample-based peak filtered. Features with ≥20% missing-values were filtered out, and probabilistic quotient normalisation applied [113]. RSD values were calculated for each *m*/*z* feature, and features in QC samples with RSD values >30% omitted from the data. Missing values imputation (k-NN) was performed and the generalised log transformation applied for multivariate data analysis.

Statistical tests (t-test, Kruskal-Wallis test, ANOVA) were performed using R and the R-Stats base package, PCA and PLS-DA conducted using MetaboAnalyst v4.0 [114]. Set enrichment analysis conducted with a java-based tool [40]. The method calculates an enrichment score of a supplied set S of variables against test data, to determine whether variables of S occur tendentially within the *m*/*z* features that are high-ranking in importance for separation of phenotype in the test data. S commonly comprises elements of a biological pathway (pathway enrichment analysis), however may contain individually defined variables. Here, S comprised *m*/*z* features with high LFC of one biological batch, which were checked for enrichment against LFC-ranked lists of *m*/*z*-values in the other biological batches. Thus, it was determined whether those *m*/*z*-values that were responsible for LFC-driven phenotype-separation in one biological batch were equally important in the other biological batches. Strength of the enrichment was calculated using NES metric (normalised enrichment score), significance determined by estimating false-discovery rate corrected *p*-values using 1000-fold permutation of the *m*/*z* features. *m*/*z* features within S deemed most impactful towards a high enrichment metric NES are referred to as the leading edge subset (LE, [40]).

### 4.5. Water Chemistry of Chlorobenzene

To measure concentrations of chlorobenzene in test medium over time, algae were incubated in vials with medium containing 14 mg/L or 24 mg/L chlorobenzene (nominal) for 12 h 15 min. At 0 h and 12 h 15 min, 100 µL (*n* = 4) was injected into 20 mL headspace vials with PTFE-lined septa (Scientific Glass Laboratories, UK) containing 9.7 mL CGM and 100 µL 0.8 M H_2_SO_4_. Samples were stored in the dark at 4 °C. One hundred µL of 10 mg/L d_5_-chlorobenzene (99 atom-%, Sigma-Aldrich) in CGM was added immediately prior to analysis (final concentration 0.1 mg/L). Headspace GC-MS was performed on an Agilent 5975B inert XL MSD using DB-624 column (30 m × 0.25 mm i.d. × 1.4 µm) and Gerstel Autosampler MPS2. Analytes were identified using target *m*/*z* = 112 and qualifier *m*/*z* = 77 (chlorobenzene); target *m*/*z* = 117 and qualifier *m*/*z* = 82 (d_5_-chlorobenzene). Sample sequence was randomised and quantification of chlorobenzene was performed relative to d_5_-chlorobenzene.

## 5. Conclusions

Firstly, we designed an alternative closed-vial chemical exposure system that allows for sufficient growth of *C. reinhardtii* (at high inoculation cell densities) for a metabolomics assay, while minimising test duration and chemical volatilisation. The latter was proven by stable exposure concentrations to chlorobenzene over the test duration. We also examined the repeatability of this *C. reinhardtii* exposure system, identifying batch variation when applying unsupervised multivariate analyses to the metabolomics data, yet reducing these unwanted effects by normalising each biological batch to its respective control data. Furthermore, we used set enrichment analysis as well as supervised multivariate analysis to demonstrate the consistency of the metabolic markers discovered in three repeat exposures to chlorobenzene. Secondly, we have demonstrated that the application of an *omics* technology to synchronised algal cell cultures grown in alternating light:dark cycles can detect characteristic metabolic phenotypes that evolve through the cell-cycle. This should benefit the interpretability of molecular data in terms of discovering early mKEs that are anchored to, and predictive of, the algal adverse phenotype of reduced growth.

## Figures and Tables

**Table 1:** ANOVA results comparing PC scores of control samples only, CB samples only, and CB samples normalised to the batch-specific controls, in each case over the three independent biological batches.

Samples Analysed by PCA (All Three Batches)	PC1	PC2	PC3
Controls only	*p* = 7.15 × 10^−6^	*p* = 0.11	*p* = 3.84 × 10^−4^
CB only	*p* = 1.72 × 10^−7^	*p* = 0.20	*p* = 0.048
CB normalized to batch-specific controls	*p* = 0.20	*p* = 0.015	*p* = 0.11

**Table 2:** Enrichment analysis applied to three metabolomics datasets following exposure of *C. reinhardtii* to chlorobenzene in independent experiments (batches 1, 2, 3). Ranked lists of *m*/*z* feature LFC were calculated (S_B1_–S_B3_ for batches 1–3) and every list was compared against every other list to determine whether S_B1_-S_B3_ were enriched with elements of the other sets. Significance of enrichment was assessed using 1000 random permutations of *m*/*z* values in LFC-ranked lists, and false-discovery rates are reported. NES = normalised enrichment score, degree of metabolite enrichment of S within dataset. LE = leading edge, subset of *m*/*z* features in S that are most impactful towards a high NES.

Metabolite Set	Metric	Batch 1	Batch 2	Batch 3
S_B1_	NES	-	1.39	1.33
	LE	-	11 features	72 features
	FDR	-	0.004	0.006
S_B2_	NES	1.41	-	1.96
	LE	53 features	-	43 features
	FDR	<0.001	-	<0.001
S_B3_	NES	1.38	2.02	-
	LE	61 features	50 features	-
	FDR	<0.001	<0.001	-

## Supplementary Materials

The following are available online at https://www.mdpi.com/2218-1989/9/5/94/s1, Figure S1: Metabolic fingerprints for pellet wash optimisation, Table S1: Unique m/z features in direct infusion mass spectra in dilution study, Figure S2: Growth rates and pH drift over 24 h growth of synchronised *C. reinhardtii* in CGM medium, Figure S3: Dose-response curve of 3,5-dichlorophenol, Figure S4: Relative standard deviation of *m*/*z* features per group between three independent repeated exposure studies, Figure S5: PCA scores plots (PC1 vs. PC3) visualising the similarities and differences between three independent repeated exposure studies.
