# Supplementary material for: Improved Algal Toxicity Test System for Robust Omics-Driven Mode-of-Action Discovery in Chlamydomonas reinhardtii
Source: Metabolites. 2019 May 10;9(5):94. doi: 10.3390/metabo9050094 (PMC6572051; doi:10.3390/metabo9050094)
Supplement: Supplementary file 1 [file metabolites-09-00094-s001.pdf]

Supplementary Materials: Improved algal toxicity test system for robust *omics*-driven mode-of-action discovery in *Chlamydomonas reinhardtii*

Stefan Schade [1], Emma Butler [2], Steve Gutsell [2], Geoff Hodges [2], John K. Colbourne [1] and Mark R. Viant [1,*]

[1] School of Biosciences, University of Birmingham, Edgbaston, Birmingham B15 2TT, UK
[2] Safety and Environmental Assurance Centre, Unilever, Colworth Science Park, Sharnbrook MK44 1LQ, UK
* Correspondence: m.viant@bham.ac.uk; Phone: +44 (0)121 414 2219

2. Results

2.1 Method optimisation of biomass harvesting and extract resuspension

Prior to applying the DIMS metabolomics method in the main study, two comparative experiments were conducted to optimise the sampling and metabolite extraction protocols.

First, the impact of the number of cell pellet washes on sample variability was assessed, characterised in multivariate group clustering and median relative standard deviation (mRSD) of all *m*/*z* features in groups of 10 technical replicates from the same biological batch culture. Cell pellets were either not washed, washed once or washed twice with 10 mL of a -20 °C 35% methanol:water solution between centrifugation steps and prior to flash freezing. The mRSD value of unwashed cell pellets was 28.08%, which decreased to 21.22% for pellets washed once, and to 16.76% for pellets washed twice. No pellet washing led to high sample variation in multivariate space (PCA scores plot; Supplementary Fig. S1). A significant difference was discovered between the three wash groups along PC1 (ANOVA, $p = 6.19 \times 10^{-9}$), capturing 47.6% variance in the dataset, but not along PC2 ($p = 0.2$), capturing only 11.2% variance. Despite the reduction in mRSD between one and two pellet washes, no substantial difference in clustering could be found between these two wash conditions, however PC2 scores (not PC1) were significantly different between one and two washing steps (t-test, $p = 0.06$).

Second, the effects of dilution of the reconstituted metabolite extracts on the detectability of unique *m*/*z* features in positive ion mode nESI DIMS analysis were characterised. Polar sample extracts of triplicate samples from the same biological batch were reconstituted in 50 µL injection solvent (neat) and diluted 3-fold, 9-fold, 27-fold and 81-fold with injection solvent. Numbers of unique features before pre-processing (after peak alignment, including blank features), and after blank- and sample filtering that retain only features found in 100% of samples were calculated (Supplementary Table S1). The neat resuspension volume yielded the highest number (4602) of unique *m*/*z* features after sample filter, with decreasing number of features with increasing dilution (3-fold = 3813, 9-fold = 2204, 27-fold = 1540, 81-fold = 694).

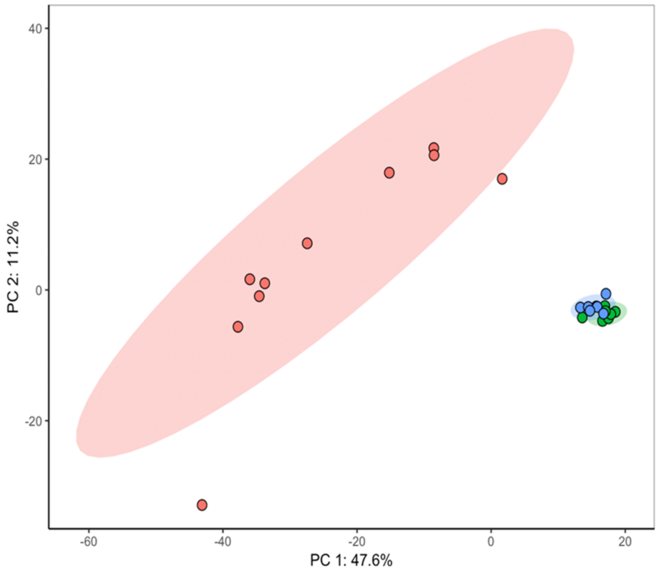

Figure S1. Metabolic fingerprints for pellet wash optimisation. A first study comprising synchronised control cultures seeded in vials was performed to assess the influence of the number of washing steps on the metabolomics data quality. Cell cultures were concentrated as above from a single stock, seeded at 7.5 x 10^5 cells/mL in 30 capped vials and incubated for 3 h. Next, 5.25 x 10^6 cells were sampled from each test vial, and harvesting performed as described above with varying number of pellet washes: None (Red), once (Blue) or twice (Green).

	After alignment, incl. blank	*After blank filter*	*After sample filter (100%)*
Neat	14115	7649	4602
3x	12986	6409	3813
9x	11563	4888	2204
27x	10008	3310	1540
81x	9591	2881	694

Table S1. Unique *m*/*z* features in direct infusion mass spectra from various degrees of dilution of injection solvent, after alignment, after blank and after 100% sample filter. A set of n = 3 algae sample pellets with 5.25 x 10^6 cells underwent metabolite extraction and dried extracts reconstituted in 50 µL injection solvent were diluted 3-, 9-, 27- and 81-fold for separate injections and comparison of detectable unique features (peaks) after processing. A reconstitution volume of 50 µL ("Neat") injection solvent was found to result in the maximal number of unique m/z features post-processing.

2.2. Validation of C. reinhardtii test system for volatile substances

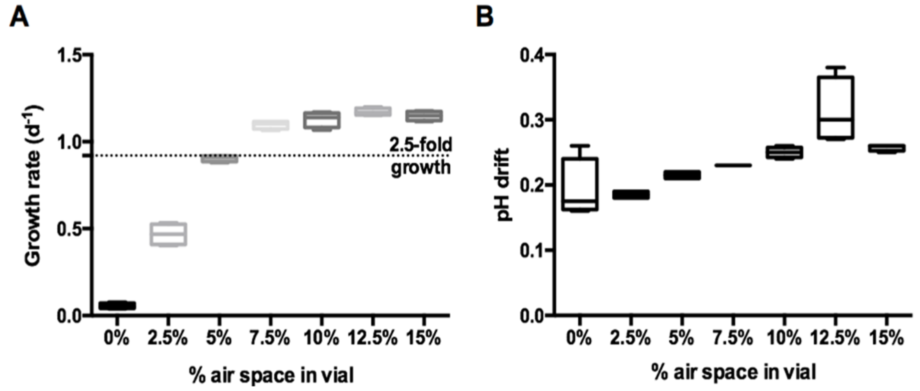

Figure S2. Growth rates (A) and pH drift (B) over 24 h growth of synchronised *C. reinhardtii* cultures in capped vials, inoculated at 7.5 x 10^5 cells/mL in CGM. 2.5-fold growth per day as validity criterion [1] is marked as dotted line.

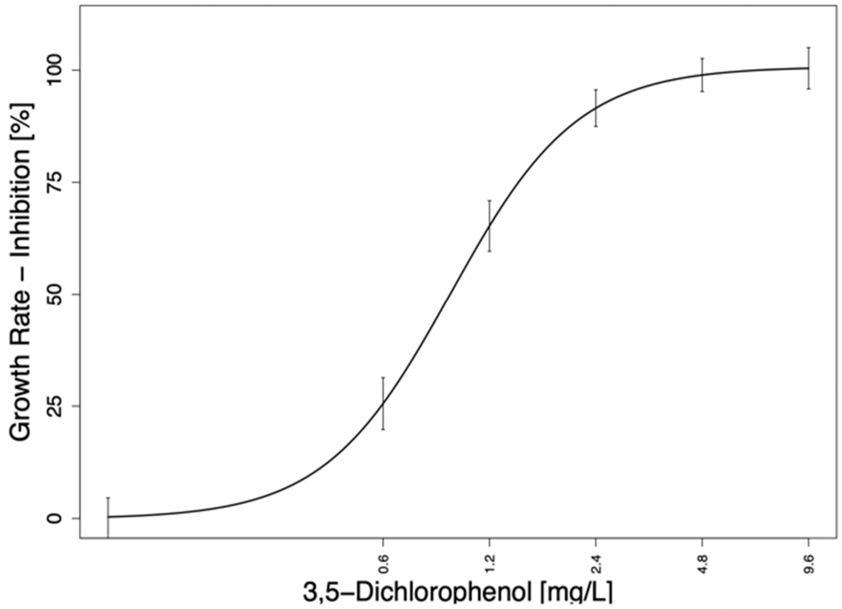

Figure S3. Dose-response curve (4 parameter log-logistic model) of 3,5-dichlorophenol, a reference substance recommended by the OECD 201 Test Guideline [1] to prove validity of the exposure system. The estimated EC_{50} = 0.93 mg/L lies well within the range of values in the literature.

2.3. Repeatability of C. reinhardtii metabolic phenotypes in test system

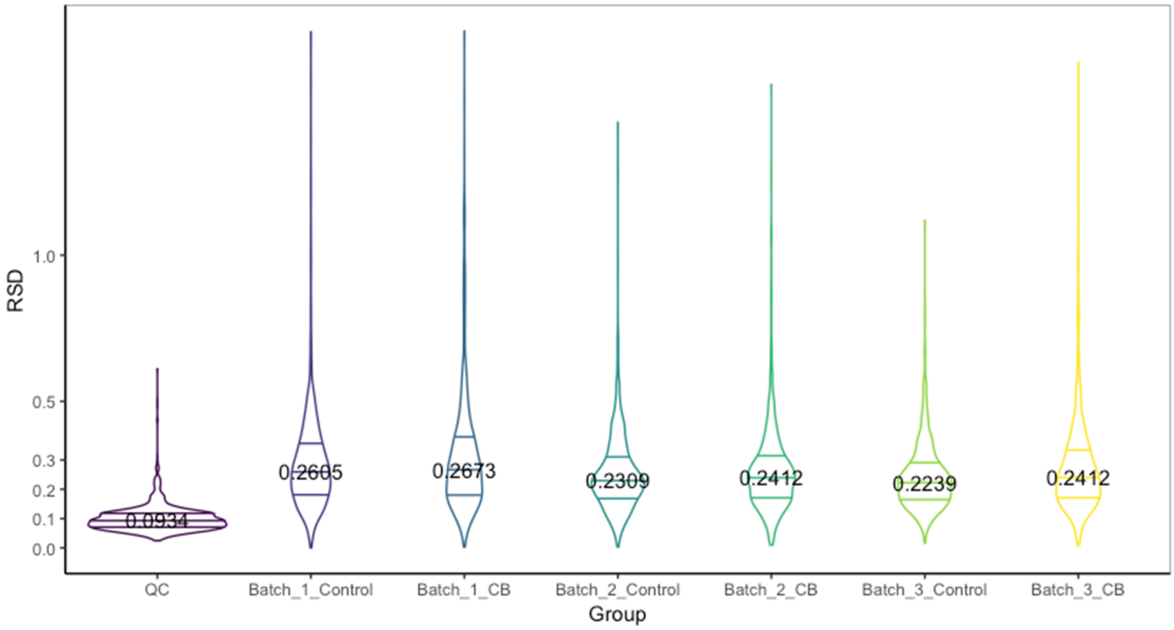

Figure S4. Relative standard deviation (RSD) of *m*/*z* features per group as well as annotated median RSD value over all features, comparing three independent repeat exposure batches. Control vs exposed (CB), n = 10 per group.

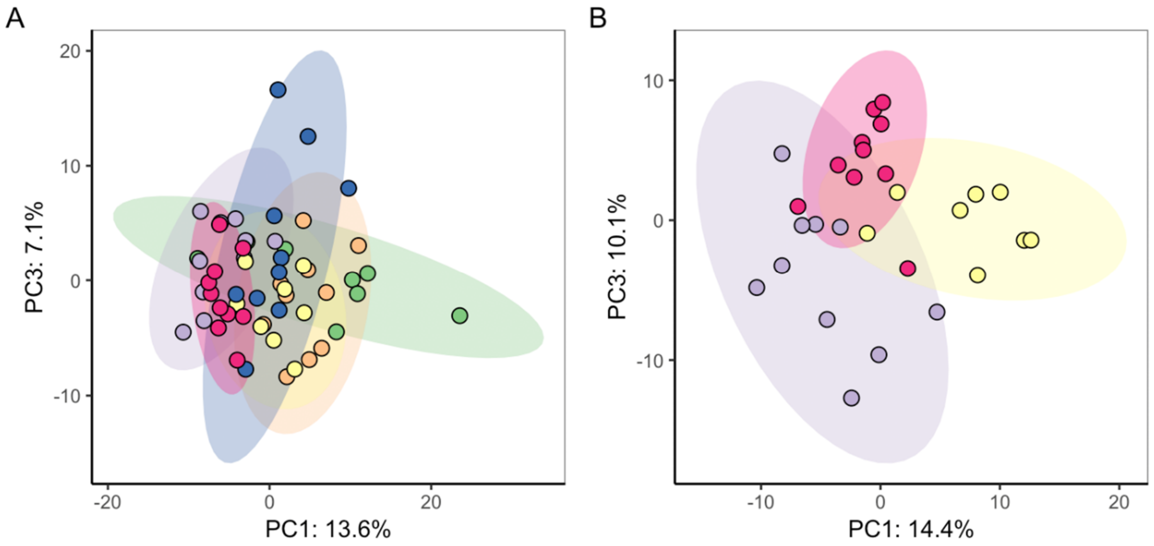

Figure S5. PCA scores plots (PC1 vs. PC3) visualising the similarities and differences between three independent repeated exposure studies (biological batches), including each control and CB group (each n = 10). (A) All six control and CB groups, across all biological batches, and (B) control groups only, across the three batches. Groups were: Biological batch 1 Control (Purple), CB (Green), batch 2 Control (Yellow), CB (Orange), and batch 3 Control (Red), CB (Blue).

References

1. OECD. Test No. 201: Alga, Growth Inhibition Test. In *OECD Guidelines for the Testing of Chemicals, Section 2: Effects on Biotic Systems*; OECD Publishing: Paris, France, 2011.
